# The impact of social media on athletes' mental health: a systematic review

**DOI:** 10.3389/fspor.2026.1876771

**Published:** 2026-08-06

**Authors:** Jaime Siaj, Diego Domínguez-Balmaseda, Antonio Vos-Saz, Iván Bonilla

**Affiliations:** 1 Department of Real Madrid Graduate School, Faculty of Medicine, Health and Sports, Universidad Europea de Madrid, Madrid, Spain; 2 Facultad de Ciencias Biomédicas y de la salud, Universidad Alfonso X El Sabio (UAX), Villanueva de la Cañada, Madrid, Spain

**Keywords:** athletes, burnout, depressive symptoms, emotional distress, mental health, social media

## Abstract

Social media use has reached almost every sport context, yet its psychological implications for athletes remain inconsistently defined. Existing research suggests potential links between social media engagement and mental health outcomes, but findings vary across athlete populations, platforms, and usage patterns. The aim of this systematic review was to synthesize empirical evidence examining the relationship between social media use and mental health outcomes in athletes, and to identify key moderators, gaps, and implications for sport psychology practice. A systematic search of EBSCOhost, PubMed, Scopus, Web of Science, and Google Scholar was conducted between October and December 2025 to identify peer-reviewed empirical studies examining social media use and mental health outcomes in athlete populations. Studies were screened according to predefined eligibility criteria, and eleven studies met the inclusion criteria and were included in the final synthesis. Data were extracted and synthesized narratively due to heterogeneity in study design, athlete samples, social media measures, and psychological outcomes. Across the included studies, social media use was frequently associated with negative mental health outcomes, including anxiety, depressive symptoms, emotional distress, body image concerns, and burnout. Passive engagement, appearance-based comparison, and problematic use patterns were more consistently linked to poorer psychological outcomes, whereas active engagement and context-dependent use showed mixed or potentially protective associations. However, given the predominance of cross-sectional designs, heterogeneity in measurement, and the still limited number of studies, these findings should be interpreted cautiously. Overall, current evidence suggests that social media use may relate to athletes' mental health in complex and context-dependent ways. The strength and direction of associations appear to vary according to usage patterns, developmental stage, and competitive level. Further longitudinal, platform-specific, and elite-focused research is required to clarify mechanisms, temporal relationships, and population-specific effects.

## Introduction

Social media (SM) has become an integral part of daily life, reshaping how individuals connect, communicate, and consume information (1). What began as a tool for staying connected has developed into a digital environment that shapes norms, expectations, and social comparison processes across social, educational, and professional settings (2). Research in the general population shows that SM use can have both positive and negative effects on well-being depending on how, why, and when individuals use these platforms. On the negative side, several studies indicate that problematic or emotionally driven patterns of use, including frequent checking, passive browsing, and tendencies to compare oneself to others online, are associated with higher levels of depression, anxiety, and stress (3, 4). These relationships appear to be explained not by the amount of time spent on social media, but by the type of engagement and the individual's sensitivity to social feedback or comparison processes (5, 6).

At the same time, social media can offer meaningful psychological benefits. Many users report increased feelings of belonging, emotional connection, and social support when they engage actively with others online or participate in communities that reflect their interests and experiences (7). Positive feedback, opportunities for self-expression, and maintaining relationships through messaging or shared content can enhance mood and support identity development, especially for younger individuals navigating important social transitions (5). Because of these contrasting outcomes, recent literature emphasises the importance of understanding social media not as inherently harmful or helpful, but as a context that can produce either effect depending on the person, the content, and the pattern of use (6).

For athletes, the situation is more complex. Their performance-based identities, public visibility, and demanding schedules mean that social media interacts with a unique set of psychological pressures. Athletes often juggle training, competition, academic or work responsibilities, and personal life while simultaneously managing expectations from coaches, teammates, and the public (8). Much of the existing empirical work has focused on student-athletes, showing both benefits and risks associated with social media use. Positive uses such as communication, emotional support, or maintaining team connection can enhance mood and a sense of belonging (9). However, negative experiences, including exposure to criticism, comparison, or performance-related commentary, have been linked to higher anxiety, stress, and poorer well-being (9). Research examining athletes has reported associations between social media use and a range of psychological outcomes, including emotional well-being, sleep, body image, and social comparison. However, the evidence remains heterogeneous, and the mechanisms through which social media may influence athlete mental health are not yet fully understood. Despite these emerging findings, several methodological limitations constrain current understanding of the relationship between social media use and athletes' mental health.

Most studies use cross-sectional, self-report designs, which prevents clear conclusions about causal direction and raises the possibility that distressed athletes simply use social media differently. Many samples are small and drawn from single teams or institutions, limiting generalisability. In addition, studies vary widely in how they define and measure social media use, with some focusing on time spent, others on specific platforms, and others on broad constructs such as engagement or problematic use (8, 9). Longitudinal and experimental studies are rare, making it difficult to understand how changes in social media habits influence well-being over time. These methodological inconsistencies mirror broader issues seen in general-population research (2, 10) and they highlight the need for more rigorous and consistent approaches when studying social media use in athletic populations.

Gender and role differences also appear to influence how athletes experience social media, with preliminary evidence suggesting that male and female athletes may use platforms differently and show different patterns of anxiety, stress, or self-esteem related to SM use (9). While most evidence comes from adolescent and collegiate samples, early findings suggest that effects may differ for elite or professional athletes. Gender and performance level may also influence how athletes experience social media. Preliminary evidence suggests that social media engagement and its psychological consequences may differ according to sex, developmental stage, and competitive level. However, most existing research has focused on adolescent and collegiate athletes, while elite and professional athletes remain substantially underrepresented. This represents an important gap, as elite performers face unique pressures related to public scrutiny, personal branding, and online visibility. Despite increasing interest in this topic, research directly examining social media's influence on the mental health of elite athletes remains limited. This represents a significant gap, as elite performers face unique pressures related to public scrutiny, branding demands, and emotional exposure in online spaces.

Given the growing and multifaceted role of social media in athletes' lives, there is a clear need to synthesise empirical evidence examining how social media use relates to athletes' mental health. Therefore, the aim of this systematic review was to synthesize the available empirical evidence examining the relationship between social media use and mental health outcomes in athletes. Specifically, the review sought to identify the psychological outcomes associated with social media use, examine factors that may influence these relationships, and highlight priorities for future research and applied practice in sport psychology.

## Methodology

### Study design

This study employed a systematic review methodology to examine the relationship between social media use and mental health outcomes among athletes. The review was conducted in accordance with the Preferred Reporting Items for Systematic Reviews and Meta-Analyses (PRISMA 2020) guidelines. A structured and transparent approach was used to identify, screen, and synthesize empirical research addressing the psychological implications of social media engagement in athletic populations.

### Search strategy (search code)

A systematic literature search was conducted using the EBSCOhost interface (including APA PsycInfo, MEDLINE Complete, CINAHL Complete, and SPORTDiscus), together with PubMed, Scopus, Web of Science, and Google Scholar. Google Scholar was used as a supplementary search tool to identify additional records not captured through database indexing. To improve reproducibility, screening was limited to the first 200 results sorted by relevance. The search strategy combined terms related to social media use, athlete populations, and mental health outcomes. Only studies published in English or Spanish were considered for inclusion. The following Boolean search string served as the basis for all database searches. Minor database-specific adaptations (e.g., field tags, subject headings, and indexing syntax) were made where necessary to accommodate the search functionality of each database while preserving the same conceptual search framework. The complete database-specific search strategies are provided in Appendix A.

Reference lists of included studies were also screened to identify additional relevant articles. Grey literature and unpublished studies were not systematically included. However, Google Scholar was used as a supplementary source to identify additional potentially relevant records. The search and screening process were conducted independently by three reviewers, with final study inclusion determined by agreement of at least two reviewers.

### Eligibility criteria

Studies were eligible for inclusion if they met the following criteria: (1) the study population consisted of athletes (including collegiate, elite, semi-elite, or competitive athletes, as well as organized team or individual sport participants); (2) the study explicitly examined social media use or social networking platforms (e.g., Instagram, TikTok, Facebook, Twitter/X, smartphone social networking applications) as an exposure or contextual factor; and (3) the study reported mental health or psychosocial outcomes. Mental health outcomes were operationally defined and included indicators of psychological well-being or ill-being such as depression, anxiety, emotional distress, psychological well-being, body image–related distress, and athlete burnout (e.g., emotional or physical exhaustion, reduced sense of accomplishment, and sport devaluation).

Both quantitative and qualitative peer-reviewed empirical studies were eligible for inclusion. Studies were excluded if they (1) did not include an athlete population, (2) did not assess social media use or social media–related experiences, or (3) focused exclusively on sport performance outcomes without examining mental health or psychosocial variables. Review articles, editorials, conference abstracts, dissertations, and non–peer-reviewed literature were also excluded.

Given the heterogeneity of study designs, outcome measures, and methodological approaches across the included studies, MMAT ratings were incorporated into the narrative synthesis to contextualize the strength and consistency of findings across studies.

### Quality assessment

Methodological quality of included studies was evaluated using the Mixed Methods Appraisal Tool [MMAT; (11)], which allows appraisal of qualitative, quantitative, and mixed-methods studies within a single framework. The MMAT assesses five methodological domains specific to each study design, including sampling strategy, measurement appropriateness, risk of nonresponse bias, confounding control, data collection procedures, and coherence between data and interpretation.

Each included study was independently assessed against the relevant MMAT criteria according to its design classification (qualitative, quantitative descriptive, or mixed methods). Studies were not excluded based on quality; rather, methodological strengths and limitations were considered during synthesis and interpretation.

Overall, methodological quality across studies was moderate. A summary of the methodological quality assessment is provided in Table 1. Most quantitative studies demonstrated appropriate measurement tools and clear research questions; however, common limitations included cross-sectional design, reliance on self-report data, and limited control for confounding variables. Qualitative studies showed strong coherence between data and interpretation but were limited by small sample sizes and context-specific recruitment. The single mixed-methods study adequately integrated qualitative and quantitative components but shared similar limitations regarding generalizability.

**Table 1:** Summary of methodological quality (MMAT).

Study	Design	Overall Quality
Barry et al. (13)	Quantitative	Moderate
Fiedler et al. (8)	Quantitative	Moderate
Staśkiewicz-Bartecka et al. (17)	Quantitative	Moderate
Bjornsen & O'Connor (18)	Quantitative	Moderate
Lin et al. (14)	Quantitative	Moderate
Pacewicz et al. (19)	Quantitative	Moderate
Smith (20)	Qualitative	Moderate–High
Hockin-Boyers et al. (21)	Qualitative	Moderate–High
Suchland et al. (16)	Mixed methods	Moderate
Yang et al. (22)	Quantitative	Moderate
Watkins et al. (15)	Quantitative	Moderate

A detailed item-level MMAT appraisal for each included study is provided in Table 1, including the two screening questions (S1–S2), the five design-specific methodological criteria (C1–C5), and the overall methodological appraisal.

## Results

### Study selection

**Figure 1:**
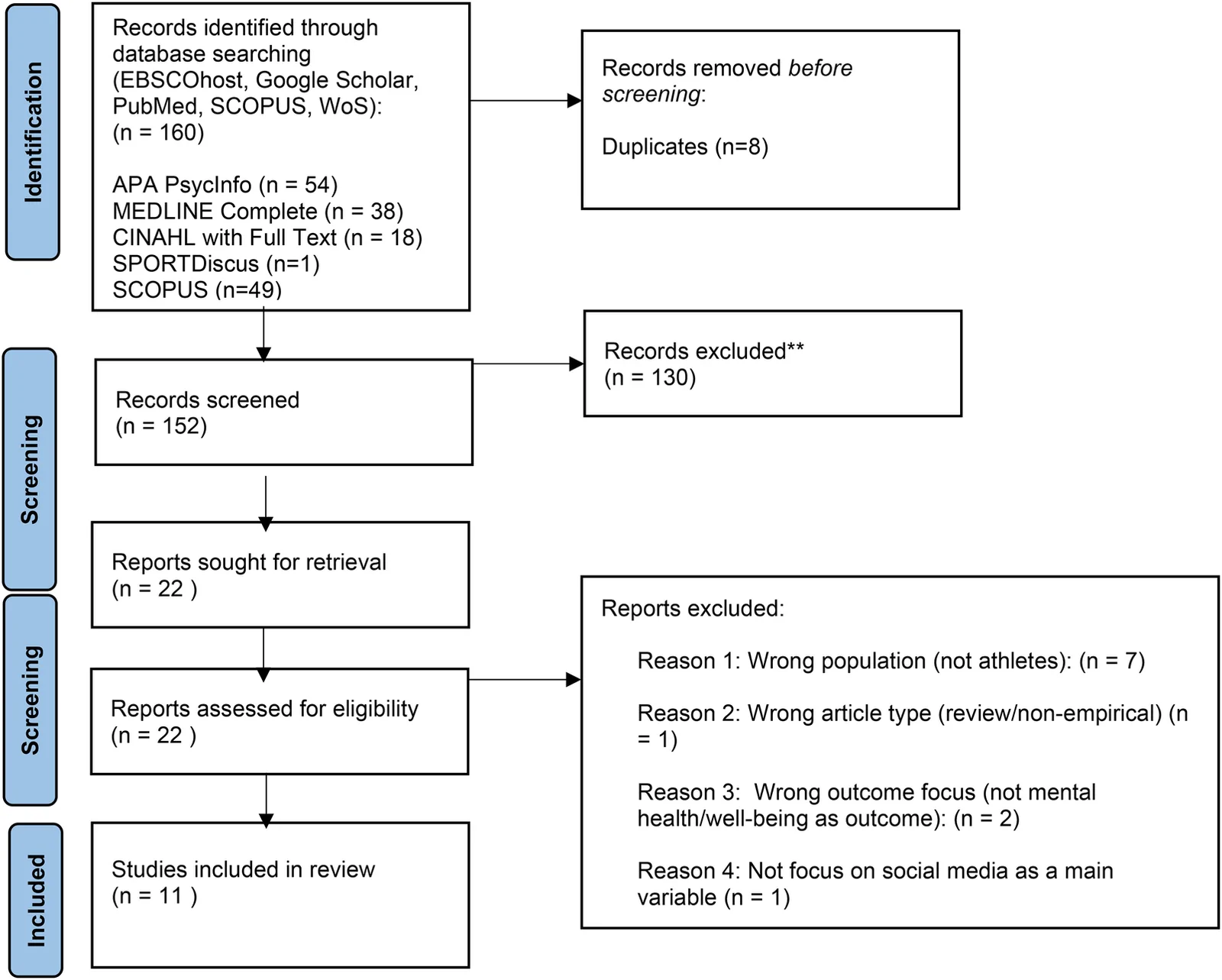
The PRISMA figure showing the steps to choose the studies for systematic review (12).

All records identified through database searching were exported and screened. A two-stage screening process was conducted: title and abstract screening followed by full-text screening. Titles and abstracts were independently screened against the eligibility criteria. The full study selection process is presented in Figure 1. Despite the broad search strategy and additional screening of reference lists, only a limited number of studies met the predefined eligibility criteria, reflecting the emerging nature of research in this area. Records were excluded at this stage if they clearly:

- Did not include an athlete population
- Did not examine social media or digital media
- Did not assess mental health or psychological outcomes

### Identification

**PRISMA CHART:**

### Study selection

The search was conducted between October 1 and 31 of December 2025, with 31 December 2025 serving as the final search date. Only studies that were publicly available and indexed in the selected databases by the final search date were considered eligible for inclusion. Database searches identified 160 records across the selected information sources. After removal of 8 duplicate records, 152 records were screened by title and abstract. Of these, 130 records were excluded because they did not meet the eligibility criteria. A total of 22 full-text reports were sought for retrieval and all were successfully retrieved and assessed for eligibility. Eleven reports were excluded after full-text assessment: seven did not include an athlete population, one was a review or non-empirical article, two did not examine mental health or well-being outcomes, and one did not focus on social media as a main variable. Eleven studies met all eligibility criteria and were included in the final qualitative synthesis.

A total of 22 articles were retained for full-text assessment, during which eligibility was evaluated according to predefined inclusion and exclusion criteria. Full-text articles were excluded if they focused exclusively on athletic performance without mental health variables, examined general populations rather than athletes, addressed digital media without a social media component, or lacked sufficient empirical data relevant to psychological well-being.

Following full-text screening, eleven studies met all eligibility criteria and were included in the final qualitative synthesis. These studies examined associations between social media use and mental health outcomes among athletes across diverse developmental stages, sport types, and competitive levels, including adolescent, collegiate, elite-pathway, and para-athletes. The final synthesis included cross-sectional quantitative studies, qualitative investigations, and one mixed-methods study. This systematic review was not prospectively registered. However, the review followed the PRISMA 2020 guidelines, and all methodological procedures were predefined and reported transparently.

### Study characteristics

The final synthesis included eleven peer-reviewed studies published between January 2022 and December 2025 examining associations between social media use and mental health outcomes among athletes across diverse competitive levels. Samples included adolescent, collegiate, recreational, elite-pathway, and para-athletes, representing both male and female participants across multiple sports. Study designs comprised eight quantitative studies (including seven cross-sectional studies and one prospective longitudinal study), two qualitative studies, and one mixed-methods study. Sample sizes ranged from 10 to 1,120 participants. The characteristics and main findings of the included studies are summarized in Tables 2 and 3.

**Table 2:** Main results or conclusions of studies on the impact of social media on athletes.

Authors (Year)/Location	Title	Athlete Population Level	Study Design	Sample Size (*N*)	Social Media Platform(s)	Social Media Variables	Mental Health Outcomes	Key Findings
Barry et al. (2024) (13) USA	Social media engagement, perceptions of social media costs and benefits, and well-being in college student-athletes	Collegiate student-athletes (multiple sports)	Cross-sectional survey	1,120 (338 male; 777 female; 5 non-binary)	Multiple platforms	Screen time; perceived costs/benefits; interference	Depression; anxiety; sleep; loneliness; self-esteem	Higher social media engagement and perceived interference were associated with poorer mental well-being outcomes.
Fiedler et al. (2024) (8) Germany	Young athletes' mental well-being is associated with smartphone social networking application usage and moderated by performance level and app type	Adolescent athletes (competitive to elite)	Cross-sectional study	53	Smartphone social networking apps	Daily usage time; excessive use	Negative affect; dysfunctional eating; sleep	Social media use was linked to negative affect and eating behaviors, mediated by social comparison and sleep quality.
Staśkiewicz-Bartecka et al. (2024) (17) Poland	Assessment of Eating Disorder Risk According to Sport Level, Sex, and Social Media Use among Polish Football Players	Male and female football players	Cross-sectional study	170	Social media (general)	Frequency of use; comparison	Eating disorder risk	Higher social media use was associated with increased eating disorder risk, particularly among female and higher-level athletes.
Bjornsen & O'Connor (2025) (18) USA	Self-disclosure of body image, eating, and emotional concerns by female collegiate athletes	Female collegiate athletes	Cross-sectional study	237	Social media (general)	Self-disclosure; body image pressure	Emotional distress; body image concerns	Social media pressure was associated with greater emotional distress and body image concerns.
Lin, et al. (2025) (14) China	The impact of social media addiction on the negative emotions of adolescent athletes	Adolescent athletes	Cross-sectional study	320 (198 male; 122 female)	Social media platforms	Social media addiction	Negative emotions; sleep disturbance	Social media addiction predicted negative emotions, mediated by appearance comparison and sleep.
Smith (2025) (20) Australia	“I want to change minds and destroy stereotypes”: Wheelchair motocross rider portrayals on Instagram	Wheelchair motocross athletes	Qualitative interviews & content analysis	10 (8 male; 2 female)	Instagram	Self-presentation; identity portrayal	Well-being pressure; identity stress	Athletes used Instagram to challenge stereotypes but experienced pressure to present inspirational identities.
Pacewicz et al. (2024) (19) USA	The toll of the scroll: A path toward burnout	Collegiate athletes	Cross-sectional study	162	Instagram	Browsing; interacting; social comparison	Athlete burnout (exhaustion, reduced accomplishment, devaluation)	Passive browsing and unfavorable social comparison were associated with higher burnout perceptions.
Hockin-Boyers et al. (2024) (21) UK	Digital pruning: Agency and social media use among female weightlifters in recovery from eating disorders	Female weightlifters	Qualitative interviews	19	Social media (multiple)	Content curation; unfollowing	Psychological well-being; recovery	Athletes actively curated social media to protect mental health during recovery.
Suchland et al. (2022) (16) USA	Effects of TikTok on the Mental Health of Men's and Women's Soccer Teams	Collegiate soccer players	Mixed methods	40	TikTok	Usage patterns	Stress; anxiety; mood	TikTok use influenced mood and stress levels differently across teams and genders.
Yang et al. (2024) (22) South Korea	Social media self-presentation and self-esteem	Adolescent athletes	Cross-sectional study	468 (244 male; 224 female)	Social media (general)	Self-presentation; responsiveness	Self-esteem	Honest self-presentation was associated with higher self-esteem, moderated by perceived responsiveness
Watkins et al. (2022) (15) USA	Social media use and mental health during COVID	NCAA Division I athletes	Prospective longitudinal study	242 (84 male; 158 female)	Social media apps	Screen time; usage	Loneliness; stress; anxiety	Increased social media use was associated with decreased loneliness, stress, and anxiety during COVID

**Table 3:** Direction of reported associations between social media use and mental health outcomes.

Study	Distress Outcomes	Body Image/Eating	Burnout	Sleep	Moderation	Overall Direction
Barry et al. (13)	Positive association (↑ distress)	–	–	Positive (↓ sleep)	–	Negative
Fiedler et al. (8)	Positive (↑ negative affect)	Positive	–	Positive	Performance level	Mixed
Staśkiewicz-Bartecka et al. (17)	–	Positive	–	–	Gender, sport level	Negative
Bjornsen & O'Connor (18)	Positive	Positive	–	–	–	Negative
Lin et al. (14)	Positive	Indirect via comparison	–	Mediator	–	Negative
Pacewicz et al. (19)	–	–	Positive	–	–	Negative
Smith (20)	Mixed	–	Identity pressure	–	–	Mixed
Hockin-Boyers et al. (21)	Protective patterns	–	–	–	–	Positive/Adaptive
Suchland et al. (16)	Mixed	–	–	–	Gender differences	Mixed
Yang et al. (22)	Positive (↑ self-esteem)	–	–	–	Responsiveness	Positive
Watkins et al. (15)	Negative (↓ distress)	–	–	–	Context (COVID)	Mixed/Adaptive

Social media platforms examined included Instagram, TikTok, smartphone-based social networking applications, and general multi-platform use. Mental health outcomes assessed across studies included depression, anxiety, stress, sleep disturbance, emotional distress, burnout, eating disorder risk, negative affect, and psychological well-being.

Across the eleven included studies, six reported predominantly negative associations, while others reported mixed or context-dependent effects, including potentially adaptive associations under specific conditions between social media engagement and mental health indicators. Three studies reported mixed findings, with platform-specific or context-dependent effects. No study reported exclusively null associations. Qualitative studies introduced adaptive or protective themes, suggesting variability depending on engagement style and agency.

Importantly, larger-sample quantitative studies [e.g., (8, 13)] demonstrated patterns consistent with smaller studies, lending moderate support to observed associations. However, given that only one prospective longitudinal study was identified and no randomized or controlled experimental studies were included, these findings should not be interpreted as evidence of causality. When considering methodological quality, no high-risk-of-bias study substantially contradicted the broader pattern of findings. However, the uniform reliance on cross-sectional self-report designs limits the certainty of directional interpretation across all domains. A study-level mapping of the inclusion criteria (population, social media exposure, and mental health outcome) for all included studies is provided in Table 2.

### Social media engagement and mental health outcomes

Across multiple quantitative studies, higher levels of social media engagement, perceived interference, or addictive patterns of use were consistently associated with poorer mental health outcomes among athlete populations. In a large sample of collegiate student-athletes, Barry et al. (13) found that greater social media engagement and perceived interference were associated with higher levels of depression, anxiety, loneliness, sleep disruption, and lower self-esteem. Similarly, Lin et al. (14) reported that social media addiction among adolescent athletes was positively associated with negative emotions, with indirect pathways through appearance-based comparison and sleep disturbance. In contrast, Watkins et al. (15) reported that increased social media use during the COVID-19 disruption period was associated with decreases in loneliness, stress, and anxiety among NCAA Division I athletes, suggesting that social media may also serve adaptive or socially supportive functions under specific circumstances.

Mixed-methods evidence further suggested that the psychological impact of social media use may vary by platform, team context, and gender. Suchland et al. (16) observed that TikTok use among collegiate soccer teams influenced mood and stress in nuanced ways, with athletes describing both motivational and emotionally taxing experiences depending on usage patterns.

### Body image, eating behaviors, and social comparison

Several studies highlighted body image-related vulnerabilities associated with social media use, particularly among female athletes and adolescent or collegiate samples. Staśkiewicz-Bartecka et al. (17) found that higher social media use was associated with increased eating disorder risk among Polish football players, with stronger associations observed among female athletes and those competing at higher sport levels. Bjornsen and O'Connor (18) similarly reported that perceived social media pressure related to body image and self-disclosure was associated with greater emotional distress among female collegiate athletes.

Lin et al. (14) further demonstrated that appearance-based social comparison functioned as a key mediator, linking social media addiction to negative emotional outcomes and sleep disturbance among adolescent athletes. Collectively, these findings suggest that visual and comparison-oriented social media environments may amplify body-related psychological stressors within specific athlete subgroups.

### Type of social media use and burnout

Evidence from collegiate athlete samples indicated that the nature of social media engagement plays an important role in psychological outcomes. Pacewicz et al. (19) found that passive social media use, such as browsing without interaction, was associated with higher perceptions of athlete burnout, including exhaustion and reduced accomplishment. In contrast, more interactive forms of engagement showed weaker or inverse associations with burnout dimensions. Importantly, unfavorable social comparison emerged as a key factor linking browsing behavior to burnout perceptions.

These findings underscore that time spent on social media alone may be insufficient to explain mental health outcomes, highlighting the relevance of engagement style and cognitive processes such as comparison.

### Identity, self-presentation, and psychological pressure

Qualitative evidence provided insight into identity-related pressures associated with social media use among athletes. Smith (20) explored Instagram portrayals of wheelchair motocross athletes and found that while social media served as a platform to challenge stereotypes and assert athletic identity, athletes also experienced pressure to present inspirational narratives, which contributed to psychological strain.

Conversely, Hockin-Boyers et al. (21) documented how female weightlifters recovering from eating disorders actively engaged in “digital pruning”, strategically curating their social media environments to protect psychological well-being. This process involved unfollowing triggering content and selectively engaging with supportive online spaces, illustrating adaptive coping strategies rather than passive exposure.

### Athlete level and performance context

Preliminary evidence suggests that performance level may influence associations between social media use and mental health outcomes; however, this conclusion should be interpreted cautiously. Among the included studies, only Fiedler et al. (8) explicitly examined performance level as a moderating variable and reported that associations between smartphone social networking application use and well-being differed across competitive levels. While these findings raise the possibility that elite or elite-pathway athletes may experience distinct digital stressors or possess different coping resources, the evidence base remains limited. No other included study systematically tested performance level as a moderator, and sample compositions varied widely across investigations. Therefore, any interpretation regarding elite-specific patterns should be considered preliminary and hypothesis-generating rather than conclusive.

Beyond gender and performance level, several studies included athlete subgroups that suggest potential intersectional differences. For example, Staśkiewicz-Bartecka et al. (17) reported stronger eating disorder risk associations among female football players and higher competitive levels, while Smith (20) highlighted identity-related pressures among wheelchair motocross athletes. However, few studies systematically examined intersectional moderators such as sport type, para-athlete status, racial/ethnic identity, or injury context.

### Summary of findings

Overall, the findings indicate that social media use is associated with both negative and positive mental health outcomes, with effects largely dependent on patterns of engagement, platform characteristics, and contextual factors. Most quantitative studies reported that higher levels of social media use, particularly when characterized by excessive exposure, passive browsing, or perceived interference, were associated with poorer mental health outcomes, including negative affect, emotional distress, burnout, sleep disturbance, and disordered eating behaviors (8, 13, 14, 17, 19). Passive consumption and unfavorable social comparison emerged as consistent mechanisms underlying these associations, especially on visually oriented platforms such as Instagram. In contrast, qualitative and mixed-methods studies highlighted potential benefits of social media use when athletes exercised agency and intentional control over their online environments, such as using social media for identity expression, stereotype resistance, or psychological protection through content curation (20, 21).

## Discussion

Although preliminary evidence suggests that performance level may influence how athletes experience social media, this conclusion should be interpreted with caution because only one included study formally examined performance level as a moderator. Rather than supporting definitive differences between elite and non-elite athletes, the available evidence highlights an important gap in the literature and emphasizes the need for future studies that directly compare athletes across competitive levels using longitudinal and sport-specific designs.

Associations between social media use are not uniformly beneficial for athletes' mental health. Instead, outcomes appear to depend on patterns of engagement, platform characteristics, and the psychosocial context in which athletes interact with social media. These patterns are consistent with social comparison processes and self-regulation frameworks commonly described in sport psychology literature. Associations between social media use and athlete mental health are not uniformly negative, as beneficial effects were reported in contexts involving honest self-presentation and socially disruptive periods such as COVID-19 (15, 22).

Since nearly all included studies employed cross-sectional designs, causal direction cannot be established. Although several studies reported mediation pathways linking problematic social media use to negative emotions through mechanisms such as social comparison and sleep disturbance [e.g., (14, 19)], reverse or reciprocal relationships are equally plausible. Athletes experiencing distress or burnout may engage more frequently in passive or maladaptive social media use, potentially reinforcing negative psychological states. Consistent with general population research suggesting that social media – well-being associations are often small, context-dependent, and bidirectional (2), the current athlete evidence is more consistent with reciprocal dynamics than with a single causal pathway.

Although gender and performance level were the most frequently examined moderators, other intersectional factors likely shape athletes' digital experiences. For example, athletes in sports with strong aesthetic components may be particularly vulnerable to appearance-based comparison processes identified in the included studies (17, 18). Similarly, qualitative findings from para-athletes suggest that social media can amplify identity-related pressures and stereotype negotiation (20). However, systematic examination of intersectional moderators including sport type, para-athlete status, racial/ethnic identity, and injury status remains limited.

Collectively, the findings suggest that the psychological consequences of social media use are best understood as dynamic interactions between individual characteristics, patterns of engagement, and sport-specific environmental demands rather than as direct effects of platform exposure alone.

### Negative mental health outcomes and risk pathways

Most quantitative studies identified associations between higher social media use and poorer mental health outcomes, including increased negative affect, emotional distress, sleep disturbance, burnout symptoms, and disordered eating behaviors (8, 13, 14, 17, 19). However, newer evidence also suggests that some forms of engagement may be associated with improved self-esteem or reduced distress under specific contextual conditions. These findings suggest that excessive or dysregulated social media use may exacerbate psychological vulnerabilities already present in athletic environments characterized by performance pressure and evaluation.

Social comparison, particularly appearance-based comparison, emerged as a central mechanism underlying these associations. Studies focusing on visually oriented platforms such as Instagram consistently linked passive browsing and exposure to idealized athletic content with burnout symptoms, body image concerns, and eating disorder risk (17, 19). Additionally, sleep disruption was identified as an important mediator between social media use and negative emotional outcomes among adolescent athletes (14), highlighting indirect pathways through which digital engagement may affect mental health.

### Positive outcomes, agency, and adaptive use

Qualitative studies offered important counterpoints by illustrating how athletes can use social media in adaptive ways. Wheelchair motocross athletes used Instagram to challenge stereotypes and construct sporting identities, albeit alongside pressure to appear inspirational (20). Similarly, female weightlifters in recovery from eating disorders described actively curating their online environments through “digital pruning” to protect psychological well-being (21).

Additional quantitative evidence also pointed to potentially beneficial effects of social media use. Yang et al. (22) found that honest self-presentation on social media was positively associated with self-esteem among adolescent athletes, particularly when perceived responsiveness from others was high. This finding further supports the view that psychological outcomes depend not only on amount of use, but also on the nature of engagement and the social context in which it occurs. Similarly, Watkins et al. (15) reported that increased social media use during COVID-19 was associated with decreased loneliness, stress, and anxiety among collegiate athletes, suggesting context-dependent effects. These findings emphasize the role of agency and intentional engagement, suggesting that how athletes use social media may be more important than how much they use it.

### Applied implications

The findings of this review suggest several preliminary implications for sport psychology practice. Given that the current evidence base is dominated by cross-sectional studies and includes only one prospective longitudinal study, the following recommendations should be interpreted as evidence-informed rather than evidence-confirmed. Where possible, recommendations are linked to findings reported across multiple studies; others are based on more limited evidence and should be considered preliminary until further research is available. Based on the empirical findings of the included studies, social media literacy interventions in sport psychology should move beyond generic screen-time reduction and instead target specific engagement patterns associated with psychological risk.

A tiered framework may include three components:

1. Assessment of Engagement Patterns

Evidence from several quantitative studies suggests that practitioners may benefit from assessing athletes' patterns of social media engagement, particularly passive browsing, perceived interference, and problematic use (13, 19). Preliminary evidence also indicates that appearance-based comparison and sleep disturbance may represent important targets for assessment among adolescent athletes (14).

2. Skill Development and Cognitive Strategies

Preliminary qualitative evidence suggests that interventions encouraging intentional content curation (e.g., “digital pruning”) and cognitive strategies to reduce maladaptive social comparison may help athletes manage potentially harmful online experiences (21). Similarly, discussions addressing identity-related pressures may be beneficial, although current evidence is derived primarily from qualitative research (20)

3. Monitoring and Contextual Adaptation

Findings reported across several quantitative studies support monitoring changes in mood, sleep, burnout, and body image concerns in relation to social media engagement (13, 17). However, recommendations regarding developmental stage and performance level remain preliminary because performance level has been directly examined in only one included study (8).

Finally, given the limited evidence involving elite athletes, practitioners working in high-performance settings should exercise caution when extrapolating findings from adolescent and collegiate samples. These recommendations should therefore be viewed as preliminary until replicated in elite athlete populations.

## Conclusion

This systematic review synthesizes emerging research examining associations between social media use and mental health outcomes among athletes. Several studies reported similar patterns linking passive browsing, appearance-based comparison, and perceived interference with poorer psychological outcomes; however, the overall evidence remains limited and heterogeneous. At the same time, some studies suggested that honest self-presentation and socially supportive use in specific contexts may relate to more adaptive outcomes, including higher self-esteem and lower distress. Additionally, conclusions regarding elite athletes are constrained by the fact that only one included study directly tested performance level as a moderator, limiting confidence in performance-specific interpretations.

At the same time, qualitative findings suggest that athletes may experience adaptive outcomes when engagement is intentional and aligned with identity expression or psychological boundary-setting. These mixed findings reinforce that social media is not inherently detrimental or beneficial but appears to function as a contextual factor that may interact with individual vulnerabilities and sport-specific pressures.

Importantly, the strength of conclusions that can be drawn from the current evidence base is limited. Most included studies employed cross-sectional designs, with only one prospective longitudinal study identified. Most included studies employed cross-sectional designs, relied on self-report measures, and varied substantially, in conceptualization and operationalization of both social media use and mental health outcomes. Therefore, causal inferences cannot be made, and reported associations should be interpreted as preliminary.

Future research employing longitudinal, experimental, and elite-focused designs is necessary to clarify temporal dynamics, identify mediating mechanisms, and determine whether observed patterns differ across performance levels.

### Limitations and future research

Several limitations should be considered when interpreting the findings of this review. First, although multiple major bibliographic databases were searched, grey literature and unpublished studies were not systematically included. Consequently, the review may be subject to publication bias, as studies reporting non-significant or null findings are less likely to be identified through conventional database searches. Second, most included studies employed cross-sectional designs, with only one prospective longitudinal study and no randomized or controlled experimental studies, limiting causal inference regarding the directionality of relationships between social media use and mental health outcomes. Third, the heterogeneity in measurement of social media use and psychological outcomes constrained direct comparison across studies. Additionally, conclusions regarding elite athletes are constrained by the fact that only one included study directly tested performance level as a moderator, limiting confidence in performance-specific interpretations. Fourth, although athlete populations varied in age, sport type, and competitive level, research explicitly examining elite and professional athletes remains limited.

The number of included studies reflects the limited and emerging evidence base on social media and athlete mental health rather than restrictive inclusion criteria. Furthermore, the limited number of platform-specific studies constrains the ability to draw firm conclusions regarding differential mental health effects across social media platforms. Although preliminary findings suggest that visually oriented platforms such as Instagram may be more strongly associated with social comparison and body image concerns, other platforms show more context-dependent effects; however, the current evidence base is insufficient for systematic comparison. Future research should adopt platform-specific designs to examine how structural features (e.g., visual emphasis, algorithmic exposure, interaction norms) may differentially influence athlete mental health outcomes. Additionally, future research should adopt intersectional study designs to better capture contextual variability in social media - mental health associations among diverse athlete populations.

Multi-wave longitudinal designs should also be used to test reciprocal pathways between social media engagement and athlete mental health. Studies should examine mediators identified in the current evidence base, including social comparison and sleep disturbance (13, 14), and moderators such as developmental stage and performance level (8). This approach would allow clearer evaluation of temporal ordering and causal mechanisms.

Future research should prioritize longitudinal and experimental designs to clarify causal mechanisms and identify temporal effects of social media engagement on mental health. Greater attention is also needed to differentiate active versus passive social media use, platform-specific effects, and the role of contextual factors such as performance level, injury status, and public visibility. Finally, intervention-based research is warranted to evaluate strategies that promote adaptive social media use and mitigate psychological risk within high-performance sport environments.

## Data Availability

The original contributions presented in the study are included in the article/Supplementary Material, further inquiries can be directed to the corresponding author.

## Appendix A

Complete database-specific search strategies:

EBSCOhost (including APA PsycInfo, MEDLINE Complete, CINAHL Complete, and SPORTDiscus),

*TX ((“social media” OR “social networking” OR “social networking sites” OR Instagram OR Facebook OR Twitter OR X OR TikTok OR YouTube OR WhatsApp OR “digital media”)*

*AND (athlete* OR “student-athlete*” OR sport* OR esports OR “competitive gaming”)*

*AND (“mental health” OR wellbeing OR “well-being” OR psychological OR stress OR anxiety OR depression OR burnout OR “emotional distress” OR “psychological distress” OR “body image” OR “eating disorder*” OR “self-esteem”))*

PubMed

((“social media”[Title/Abstract] OR “social networking”[Title/Abstract] OR Instagram[Title/Abstract] OR Facebook[Title/Abstract] OR Twitter[Title/Abstract] OR TikTok[Title/Abstract] OR YouTube[Title/Abstract] OR WhatsApp[Title/Abstract]) AND (athlete*[Title/Abstract] OR “student-athlete*”[Title/Abstract] OR sport*[Title/Abstract]) AND (“mental health”[Title/Abstract] OR wellbeing[Title/Abstract] OR anxiety[Title/Abstract] OR depression[Title/Abstract] OR stress[Title/Abstract] OR burnout[Title/Abstract] OR “body image”[Title/Abstract] OR “self-esteem”[Title/Abstract]))

Scopus

TITLE-ABS-KEY((“social media” OR “social networking” OR Instagram OR Facebook OR Twitter OR X OR TikTok OR YouTube OR WhatsApp OR “digital media”) AND

(athlete* OR “student-athlete*” OR sport*) AND (“mental health” OR wellbeing OR psychological OR stress OR anxiety OR depression OR burnout OR “body image” OR “self-esteem”)

Web of Science Core Collection

TS = ((“social media” OR “social networking” OR Instagram OR Facebook OR Twitter OR X OR TikTok OR YouTube OR WhatsApp OR “digital media”)

AND (athlete* OR “student-athlete*” OR sport*) AND (“mental health” OR wellbeing OR psychological OR stress OR anxiety OR depression OR burnout OR “body image” OR “self-esteem”))

Google Scholar

Search terms

“social media” athletes mental health

“social networking” athlete anxiety

Instagram athlete wellbeing

TikTok athlete mental health

Screening was limited to the first 200 records sorted by relevance.
